# Excretion of mephedrone and its phase I metabolites in urine after a controlled intranasal administration to healthy human volunteers

**DOI:** 10.1002/dta.3214

**Published:** 2022-01-11

**Authors:** Joanna Czerwinska, Mark C. Parkin, Claire George, Andrew T. Kicman, Paul I. Dargan, Vincenzo Abbate

**Affiliations:** ^1^ Department of Analytical, Environmental and Forensic Sciences King's College London London UK; ^2^ Toxicology Department Eurofins Forensic Services Feltham UK; ^3^ Toxicology Department, Abbott Toxicology Ltd Alere Toxicology (now part of Abbott) Oxfordshire UK; ^4^ Clinical Toxicology, Faculty of Life Sciences and Medicine King's College London London UK; ^5^ Clinical Toxicology Guy's and St Thomas' NHS Foundation Trust and King's Health Partners London UK

**Keywords:** intranasal administration, mephedrone, metabolites, urine

## Abstract

Mephedrone is a stimulant drug structurally related to cathinone. At present, there are no data available on the excretion profile of mephedrone and its metabolites in urine after controlled intranasal administration to human volunteers. In this study, six healthy male volunteers nasally insufflated 100 mg of pure mephedrone hydrochloride (Day 1). Urine was collected at different timepoints on Day 1 and then on Days 2, 3 and 30. Samples were analysed for the presence of mephedrone and its metabolites, namely, dihydro‐mephedrone, nor‐mephedrone (NOR), hydroxytolyl‐mephedrone, 4‐carboxy‐mephedrone (4‐carboxy) and dihydro‐nor‐mephedrone (DHNM), by a validated liquid chromatography‐tandem mass spectrometry method. All analytes were detected in urine, where 4‐carboxy (C_max_ = 29.8 μg/ml) was the most abundant metabolite followed by NOR (C_max_ = 377 ng/ml). DHNM was found at the lowest concentrations (C_max_ = 93.1 ng/ml). Analytes exhibited a wide range of detection windows, but only 4‐carboxy and DHNM were detectable in all samples on Day 3, extending the detection time of mephedrone use. Moreover, mephedrone had a mean renal clearance of 108 ± 140 ml/min, and 1.3 ± 1.7% of unchanged parent drug was recovered in urine in the first 6 h post administration. It is hoped that this novel information will be useful in future studies involving mephedrone and other stimulant drugs.

## Graphical abstract

Mephedrone and its phase I metabolites exhibited a wide range of detection windows, but 4‐carboxy‐mephedrone and dihydro‐nor‐mephedrone were detectable in all samples on Day 3, extending the detection time of mephedrone use.

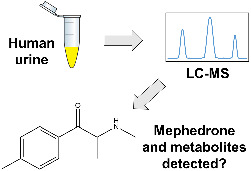

## 1 INTRODUCTION

Mephedrone (4‐methylmethcathinone) has been one of the most popular synthetic cathinones used for recreational purposes, although over the past 5 years or so, its prevalence as a ‘club drug’ appears to have waned in the UK as 3,4‐methylenedioxymethamphetamine (MDMA) has once again become more widely available. Mephedrone is typically sold as a white crystalline powder.^1^ Even though the powder can be dissolved in water prior to oral/rectal use or injection, most mephedrone users report ‘snorting’ (nasal insufflation) to be the most common route of administration.^2^, ^3^

Metabolism of mephedrone has been previously investigated in vitro^4^, ^5^ and in vivo, both in animal models^6^ and in humans.^7^ Main phase I metabolites are produced by N‐demethylation of the secondary amine, reduction of the ketone moiety to the hydroxyl group and oxidation of the tolyl moiety. Hepatic cytochrome P450 2D6 (CYP2D6) has been shown to be the main enzyme responsible for the metabolism of mephedrone in humans, with only negligible contribution from other cytochrome P450 enzymes.^4^

To date, only two controlled human administration studies have investigated the distribution of mephedrone and some of its metabolites in urine. In both studies, mephedrone was administered orally, and samples were analysed by liquid chromatography‐tandem mass spectrometry (LC‐MS/MS)^8^ or gas chromatography–mass spectrometry (GC‐MS).^9^ These studies found 4‐carboxy‐mephedrone to be the most abundant metabolite, reaching concentrations roughly 10 times higher than mephedrone itself. In addition, mephedrone showed low urinary recovery, with only about 1.15% of total administered dose being recovered following LC‐MS/MS analysis^8^ and 15.4 ± 8.4% following GC‐MS analysis.^9^ In a different study, Olesti et al.^10^ have shown urinary recovery of mephedrone and its metabolites to be proportional to the administered doses. Even though nasal insufflation is probably the most common way of using mephedrone, there are no data regarding the urinary elimination of mephedrone and its metabolites following this route of administration as part of a controlled study.

**FIGURE 1:**
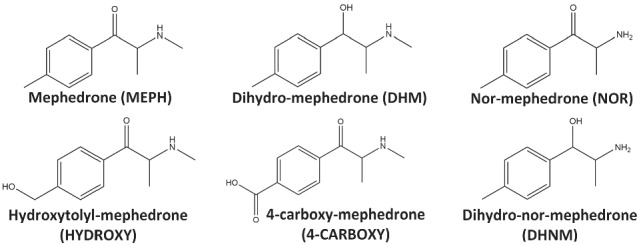
Structures of mephedrone and five of its phase I metabolites

Herein, we report for the first time the results of a study where urine samples were quantitatively analysed for mephedrone and five of its phase I metabolites (Figure 1) after nasal insufflation of 100 mg of pure mephedrone hydrochloride by six volunteers under controlled conditions. Pharmacokinetic (PK) data gained from analysis of whole blood and plasma collected in the same study have been presented previously.^11^, ^12^

## 2 MATERIALS AND METHODS

### 2.1 Reagents and standards

A detailed description of materials, consumables and reference standards has been published elsewhere.^13^ Briefly, internal standards and reference standards were from Sigma‐Aldrich (Dorset, UK) or LGC Standards (Bury, UK). Mephedrone hydrochloride used for the administration was purchased from Chiron (Trondheim, Norway). As described before, dihydro‐nor‐mephedrone was synthesised in‐house,^13^ impurities not being detected, as analysed by high resolution mass spectrometry and nuclear magnetic resonance. More information about its purity can be found in the supporting information (Section S4).

### 2.2 Blank matrix

Urine donated by drug‐free volunteers was collected into polyethylene Nalgene® bottles. Ethical approval for the collection of drug‐free matrix was granted by the Research Ethics Committee at King's College London (HR 16/17 4237).

### 2.3 Volunteer administration study and sample collection

Ethical approval for the study was obtained from the Riverside National Research Ethics Service (16/LO/1342). Details of the controlled mephedrone administration have been published elsewhere.^11^, ^12^, ^14^ In short, occasional users of mephedrone or other stimulant drugs were enrolled into the study. Volunteers were supervised throughout the study and were drug‐free 1 week before the mephedrone administration, which was verified by urine analysis by a fully validated ultra‐high‐performance liquid chromatography‐high‐resolution mass spectrometry method. Six healthy male volunteers nasally insufflated 100 mg of mephedrone hydrochloride supplied as a racemic mixture (purity: 96.3 ± 0.5%). Urine samples were collected into polyethylene Nalgene® bottles at −10 min (0 h, before administration) and 6 h on Day 1 and then at Days 2, 3 and 30. Urine samples were also collected between −10 min and 6 h on Day 1 if a participant felt the need to pass urine. Urine samples collected after mephedrone administration were not screened for other drugs (of abuse); however, participants were asked not to use any other substances until the end of the study. The volume of excreted urine at each collection timepoint was recorded. Urine samples were immediately stored at −20°C and were analysed within 2 months from the collection day. Participants in this study were not genotyped for CYP2D6 polymorphism.

### 2.4 Working solutions

Working solutions used for the preparation of the calibration curve were prepared in MeOH:water (50:50 v/v) at 15, 50, 125, 250, 500, 1000 and 1250 ng/ml for DHM, NOR and DHNM and at 40, 100, 200, 250, 500, 1000 and 1250 ng/ml for MEPH, HYDROXY and 4‐carboxy. Working solutions used for the preparation of the quality control (QC) samples at low (Low), medium (Med) and high (High) levels were made in MeOH:water (50:50 v/v) at 25, 250 and 1000 ng/ml for DHM, NOR and DHNM and at 50, 250 and 1000 ng/ml for MEPH, HYDROXY and 4‐carboxy. Internal standard (IS) solution containing MEPH‐d_3_ and DHM‐d_3_ at 50 ng/ml was prepared in MeOH:water (50:50 v/v).

### 2.5 Calibration standards and quality control samples

Matrix‐matched calibration standards containing DHM, NOR and DHNM at 0.6, 2, 5, 10, 20, 40 and 50 ng/ml, and MEPH, HYDROXY and 4‐carboxy at 1.6, 4, 8, 10, 20, 40 and 50 ng/ml were prepared by the addition of an appropriate volume of the working solution to urine. QC Low (1 ng/ml for DHM, NOR and DHNM and 2 ng/ml for MEPH, HYDROXY and 4‐carboxy), QC Med (10 ng/ml for all analytes) and QC High (40 ng/ml for all analytes) were prepared by the addition of an appropriate volume of the working solution to urine.

Calibration standards and QCs were prepared fresh on the day of sample analysis.

### 2.6 Sample preparation

A modified solid phase extraction (SPE) method developed for the extraction of mephedrone and its metabolites from human plasma (published elsewhere^13^) was used. Briefly, 250 μl of urine was extracted. Where dilution was required, samples were diluted 1 in 100 or 1 in 1000 in the blank matrix alongside three additional QCs prepared in the same manner. After SPE, samples were dried under nitrogen and were reconstituted with 100 μl of 0.1% formic acid in acetonitrile:water (10:90 v/v).

### 2.7 Instrumentation

Details of the analytical method and MS conditions have been described before.^13^ Briefly, extracted samples were analysed by LC‐MS/MS on a Waters Xevo TQ‐S triple quadrupole mass spectrometer (Waters, UK) coupled to a Waters Acquity ultra performance liquid chromatograph system (Waters, UK). Electrospray ionisation operated in positive ion mode. Retention times, selected reaction monitoring transitions and optimised collision energies are presented in Table S1. Chromatographic separation was performed on a 2.1 mm × 150 mm Selectra® column containing a 1.8 μm pentafluorophenylpropyl phase (UCT, US).

### 2.8 Data analysis

Total mephedrone eliminated in urine was calculated for each participant by multiplying mephedrone concentration in each urine sample by the urinary volume collected at each timepoint. Renal clearance was calculated by dividing the total amount of mephedrone excreted in urine by the area under the plasma concentration‐time curve (up to 6 h post administration) reported previously.^11^ The data set at each time point for the concentrations of mephedrone and its metabolites was normally distributed according to the Shapiro‐Wilk normality test performed in GraphPad Prism (version 7.0).

### 2.9 Validation procedure

Validation experiments investigated selectivity, linearity, inter‐ and intra‐day accuracy and precision, lowest limit of quantification (LLOQ), limit of detection (LOD), recovery, matrix effect, dilution integrity and carryover. Both methods were validated according to the Food and Drug Administration guidelines^15^ and recommendations published by Peters et al.^16^ Descriptions of the validation experiments can be found in the supporting information (Section S2).

## 3 RESULTS

### 3.1 Method validation

The results of method validation in urine are detailed in the supporting information (Section S3). Briefly, intra‐ and inter‐day precision was within ±15% of the true value. Mean linearity of *r* ^2^ > 0.996 was achieved for all analytes in the validation runs. Carryover was not observed. Matrix effect was within ±20% at both QC levels for all analytes, except for HYDROXY which was suppressed by 34.4 ± 7.4% at QC Low and by 32.6 ± 4.6% at QC High.

After 105 days of storage at −20°C at QC Low and QC High, all analytes were within ±15% of their initial concentration, except for DHNM at QC Low as well as HYDROXY and 4‐carboxy at QC High which lost 61.2 ± 2.9%, 33.4 ± 4.1% and 43.2 ± 8.9%, respectively. All analytes were also stable following six freeze/thaw cycles, except 4‐carboxy at QC High and DHNM at QC Low, which lost 17.2 ± 5.5% and 18.8 ± 5.3%, respectively.

### 3.2 Concentrations of mephedrone and its metabolites in urine

**FIGURE 2:**
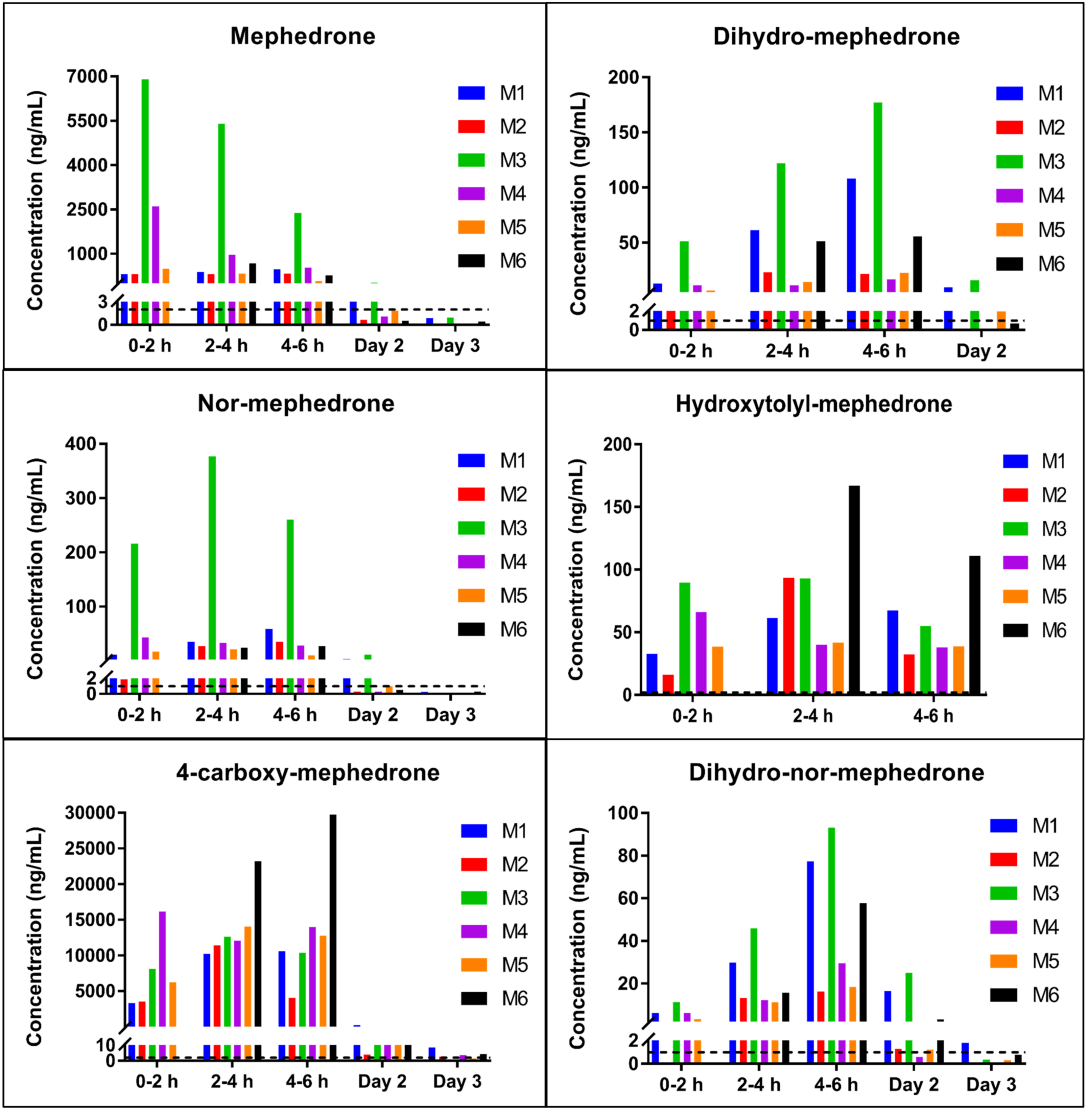
Concentration of mephedrone and its metabolites in urine collected from M1–M6; the dashed line shows the LLOQ. Concentrations shown below the LLOQ cannot be reliably quantified, and therefore, those values are only indicative. Where data for Day 2 or 3 is not shown, analytes were not detected at these timepoints [Colour figure can be viewed at wileyonlinelibrary.com]

Concentrations of mephedrone and its metabolites in urine collected from six participants (referred to here as M1–M6) are shown in Figure 2. Where data for Day 2 or 3 are not shown, analytes were not detected at these timepoints. A urine sample was not provided in the first 2 h post administration by M6; thus, no data are available for that time period for this participant. Analytes were not detected in the pre‐administration urine samples or in samples collected 30 days post administration (data not shown).

4‐carboxy reached the highest concentrations in urine (C_max_ = 29.8 μg/ml), followed by mephedrone (C_max_ = 6.98 μg/ml) and NOR (C_max_ = 377 ng/ml). DHM (C_max_ = 177 ng/ml) and HYDROXY (C_max_ = 167 ng/ml) were detected at lower concentrations, whereas DHNM was found at the lowest concentrations (C_max_ = 93.1 ng/ml). The highest concentrations of mephedrone, DHM, NOR and DHNM between 0 and 6 h were detected in M3, which showed considerably higher mephedrone and NOR concentrations compared to other participants.

Analytes exhibited a wide range of detection windows. Mephedrone was present in all participants on Day 2 and in three participants (M1, M3 and M6) on Day 3. DHM was detected in all participants except M4 on Day 2 and was undetectable on Day 3. NOR, 4‐carboxy and DHNM were all detectable in all participants on Day 2, with 4‐carboxy and DHNM also being detectable in all participants on Day 3. HYDROXY was present in samples up to 6 h but declined to undetectable levels on Day 2 in all participants.

### 3.3 Urinary recovery

Urinary recovery of mephedrone relative to the administered dose of 100 mg in the first 6 h post administration is shown in Table 1. Only 1.3 ± 1.7% of unchanged mephedrone was recovered in urine. The highest urinary recovery of 4.8% (27 μmol) was seen in M3, whereas the lowest urinary recovery of 0.2% (0.9 μmol) was reported for M6. Standard deviation (SD) presented in Table 1 was larger than the mean due to a notably higher percentage of recovered mephedrone in M3.

**TABLE 1:** Urinary recovery of mephedrone relative to the administered dose of 100 mg (calculated from the time of drug administration up to 6 h)

Participant	Mephedrone urinary recovery (%)	Mephedrone urinary recovery (μmol)
M1	0.8	4.3
M2	0.6	3.4
M3	4.8	27
M4	1.2	6.9
M5	0.6	3.1
M6	0.2	0.9
Mean	1.3	7.7
SD	1.7	9.9

Urinary recovery of mephedrone metabolites expressed as a percentage of the total dose is presented in Table 2. 4‐carboxy, which reached the highest concentrations in urine, had approximately 10 times higher urinary recovery compared to mephedrone.

**TABLE 2:** Minimum, maximum and mean urinary recoveries (% of the total dose) for mephedrone metabolites calculated from the time of drug administration up to 6 h (*n* = 6)

Analyte	Mean recovery (%) ± SD	Minimum recovery (%)	Maximum recovery (%)
DHM	0.1 ± 0.01	0.01	0.2
NOR	0.1 ± 0.1	0.01	0.3
HYDROXY	0.1 ± 0.02	0.04	0.6
4‐carboxy	12 ± 2.2	8.8	16
DHNM	0.1 ± 0.02	0.01	0.1

### 3.4 Renal clearance

Renal clearance, presented in Table 3, was calculated for all analytes and participants for the first 6 h post administration. HYDROXY displayed the greatest renal clearance (507 ± 226 ml/min), followed by 4‐carboxy (349 ± 133 ml/min) and DHNM (252 ± 294 ml/min). NOR had the lowest renal clearance of 54 ± 67 ml/min. M6 had a considerably smaller renal clearance compared to other participants.

**TABLE 3:** Summary of renal clearance (ml/min) calculated for all analytes and participants based on the data collected up to 6 h post mephedrone administration

Participant	MEPH	DHM	NOR	HYDROXY	4‐carboxy	DHNM
M1	56	294	35	701	423	17
M2	41	134	24	443	359	320
M3	383	378	187	601	435	640
M4	117	125	45	671	335	ND
M5	44	94	28	540	448	ND
M6	5.6	27	2.2	87	92	31
Mean	108	175	54	507	349	252
SD	140	132	67	226	133	294

Abbreviation: ND, not determined, because DHNM was not detected in plasma samples from M4 and M5.

## 4 DISCUSSION

4‐carboxy and DHNM were detectable in urine samples on Day 3, extending detection time of mephedrone use. In the previous study, where 150 mg of mephedrone was given orally to six healthy male volunteers, DHM, NOR and 4‐carboxy (DHNM was not included in the study) were detected in urine for up to 48 h post mephedrone administration (samples were not collected after 48 h).^8^ The ability to detect metabolites of mephedrone up to 3 days following administration provides a valuable tool from a forensic casework perspective. In those cases where mephedrone is found in urine, at or around the limit of detection, the detection of 4‐carboxy and/or DHNM adds reassurance that the drug has been administered. Likewise, detecting the presence of phase II metabolites of mephedrone is of evidential value, as a number have been reported previously.^5^, ^7^, ^17^ Because our excretion study was orientated on quantification, it was confined to the analysis of mephedrone and its phase I metabolites, as phase II standards were (and still are) not commercially available. This remains the case for conjugates of metabolites of many new psychoactive substances because synthesis is often challenging and expensive, and commercially not viable to undertake in anticipation of limited demand for such products.

From the total administered dose of 100 mg, only 1.3 ± 1.7% (1.4 mg or 7.7 μmol) of mephedrone was recovered in urine in a 6 h period. That is in close agreement with a previously reported total urinary recovery of 1.2 ± 0.3% (9.5 ± 2.9 μmol) calculated from the analysis of urine samples collected continuously for 48 h following oral administration of 150 mg of mephedrone hydrochloride.^8^ In our study, urine samples were not collected between the 6 h timepoint on Days 1 and 2, where small amounts of mephedrone were still likely excreted. Therefore, the 1.3 ± 1.7% urinary recovery does not represent total urinary recovery.

Mephedrone was rapidly eliminated with mean renal clearance of 108 ± 140 ml/min (6.5 ± 8.4 L/h). This is in agreement with the previously reported renal clearance of 5.6 ± 2.6 L/h for mephedrone which was based on the analysis of urine samples collected over a 24 h period following oral administration of 150 mg of mephedrone hydrochloride.^9^

Even though the PK blood profile of M3 (published elsewhere^11^) was similar to that of M1–M5, considerably higher concentrations of mephedrone and NOR were detected in the urine samples collected from this participant. Furthermore, the recovery of mephedrone was roughly an order of magnitude higher in M3 than that found in the other participants. On the other hand, M6 which showed notable difference in the PK profile in blood^11^ (most likely due to an altered activity of CYP2D6) had the lowest renal clearance.

According to a review describing clinical cases of mephedrone intoxication, mean urinary concentration of mephedrone was found to be 50–476 ng/ml (range: 1–198 000 ng/ml).^18^ However, in many of these cases, the exact mephedrone dose was unknown, and the route of administration varied between cases. In our study, mephedrone concentrations ranged between 1.8 and 6898 ng/ml (mean: 1001 ng/ml). In other controlled human administration studies, urinary concentrations of mephedrone over a 4 h period were 298 ng/ml (after a 50 mg oral dose), 845 ng/ml (after a 100 mg oral dose) and 2,824 ng/ml (after a 150 mg oral dose).^18^ To our knowledge, only one study so far has reported the presence of mephedrone metabolites in a urine sample collected from a forensic traffic case in Denmark. DHM, NOR, HYDROXY and 4‐carboxy were all detected but concentrations were not provided, except for HYDROXY which was found at 40 μg/kg.^4^

## 5 CONCLUSION

A fully validated method for the simultaneous quantification of mephedrone and five of its phase I metabolites in human urine has been developed and applied to the analysis of samples from a human controlled administration study in healthy volunteers. Following intranasal insufflation, all analytes were detected in urine, where 4‐carboxy reached the highest concentration. 4‐carboxy and DHNM were the only metabolites detectable in all urine samples on Day 3 post administration, extending the detection time of mephedrone use.

## Supporting information


**Table S1.** The retention time, SRM transitions and collision energy for each ion * denotes a quantifying transition ** denotes dehydrated precursor ions
**Table S2.** LOD, LLOQ, calibration range and calibration parameters for mephedrone and its metabolites in human urine
**Table S3.** Precision and accuracy at QC Low, QC Med and QC High for all analytes in human urine; * average value of 18 measurements over 3 days
**Table S4.** Analyte recovery and matrix effect at QC Low and QC High in human urine
**Table S5.** Dilution integrity (1 in 100) for mephedrone and its metabolites in human urine
**Table S6.** Dilution integrity (1 in 1000) for mephedrone and its metabolites in human urine
**Table S7.** Long term and freeze–thaw stability of mephedrone and its metabolites in human urine following storage at −20°C
**Figure S1.** ^1^H NMR spectrum of DHNM

## Data Availability

Data available on request due to privacy/ethical restrictions.
