# Supplementary material for: Excretion of mephedrone and its phase I metabolites in urine after a controlled intranasal administration to healthy human volunteers
Source: Drug Test Anal. 2022 Jan 11;14(4):741–6. doi: 10.1002/dta.3214 (PMC9306721; doi:10.1002/dta.3214)
Supplement: Supplementary file 1 — Table S1. The retention time, SRM transitions and collision energy for each ion * denotes a quantifying transition ** denotes dehydrated precursor ions Table S2. LOD, LLOQ, calibration range and calibration parameters for mephedrone and its metabolites in human urine Table S3. Precision and accuracy at QC Low, QC Med and QC High for all analytes in human urine; * average value of 18 measurements over 3 days Table S4. Analyte recovery and matrix effect at QC Low and QC High in human urine Table S5. Dilution integrity (1 in 100) for mephedrone and its metabolites in human urine Table S6. Dilution integrity (1 in 1000) for mephedrone and its metabolites in human urine Table S7. Long term and freeze–thaw stability of mephedrone and its metabolites in human urine following storage at −20°C Figure S1. 1H NMR spectrum of DHNM [file DTA-14-741-s001.pdf]

Excretion of mephedrone and its phase I metabolites in urine after a controlled intranasal administration to healthy human volunteers

Joanna Czerwinska [a], Mark C. Parkin [a, b], Claire George [c], Andrew T. Kicman [a], Paul I. Dargan [e, f], Vincenzo Abbate [a, *]

[a] Department of Analytical, Environmental and Forensic Sciences, King's College London, London, UK

[b] Eurofins Forensic Services, Toxicology Department, Teddington, UK

[c] Alere Toxicology (now part of Abbott), UK

[e] Clinical Toxicology, Faculty of Life Sciences and Medicine, King's College London, London, UK

[f] Clinical Toxicology, Guy's and St Thomas' NHS Foundation Trust and King's Health Partners, London, UK

*Correspondence to: Dr Vincenzo Abbate, King's College London, Analytical, Environmental and Forensic Sciences, 150 Stamford Street, London, SE1 9NH, UK

E-mail: vincenzo.abbate@kcl.ac.uk

Keywords: mephedrone; metabolites; urine; intranasal administration

1 LC-MS details

Table S1 shows retention times, selected reaction monitoring (SRM) transitions and optimised collision energies.

Table S1. The retention time, SRM transitions and collision energy for each ion
** denotes a quantifying transition ** denotes dehydrated precursor ions*

Analyte	Retention time (min)	Precursor ion (m/z)	Product ion (m/z)	Collision energy (V)	Internal standard
**MEPH**	5.85	160.4 **	145.1*	15	MEPH-d_3
			144.1	33	
			91.1	28	
**MEPH-d_3**	5.85	163.4 **	148.4	19	
**DHM**	5.38	162.4 **	147.3*	19	DHM-d_3
			131.4	17	
			91.3	26	
**DHM-d_3**	5.38	165.4 **	150.3	18	
**NOR**	5.00	146.0 **	131.1	25	MEPH-d_3
			130.1*	25	
			119.0	15	
**HYDROXY**	1.98	194.1	158.1	17	DHM-d_3
			146.0*	17	
			131.1	23	
**4-carboxy**	2.06	208.0	146.0*	13	MEPH-d_3
			144.1	28	
			130.1	31	
**DHNM**	4.45	148.1 **	131.1*	13	MEPH-d_3
			116.2	23	
			91.1	25	

2 Validation procedures

Validation experiments determined selectivity, linearity, inter- and intra-day precision and accuracy, limit of detection (LOD), lowest limit of quantification (LLOQ), recovery, matrix effect, carryover, dilution integrity and stability.

2.1 Selectivity

Selectivity was assessed by analysing six blank urine samples collected from drug-free female (n=3) and male (n=3) donors.

2.2 Linearity

Matrix-matched calibration curve was prepared by spiking drug-free urine with appropriate working solutions containing the analytes. Each calibration standard was required to be within ± 15% of its target concentration, except at the LLOQ where ± 20% variation was allowed. The upper level of quantification (ULOQ) was defined as the highest concentration of the calibration standard. The correlation coefficient (r^2) of the curve had to be at least 0.990. A linear regression model with a weighting of 1/x was applied to all calibration curves.

2.3 LOD and LLOQ

The LOD in a matrix was defined as the lowest concentration where all three ions (two qualifiers and one quantifier) were present with a signal-to-noise ratio equal to or greater than 3. The LLOQ was defined as the lowest concentration at which analytes could be quantified with an acceptable precision and accuracy. The ULOQ was determined as the highest concentration of the calibration curve, which could be determined with an acceptable accuracy and precision without saturating the detector.

2.4 Precision and accuracy

Intra-day (n=6) and inter-day (n=3) precision and accuracy was determined by employing quality control (QC) samples spiked at low (Low), medium (Med), and high (High)

concentrations. Intra-day precision was calculated using six replicates obtained on the same day which were expressed as a coefficient of variation (%CV). Accuracy was calculated by dividing the mean measured concentration at each QC level by the theoretical spiked concentration and was expressed as a percentage of the theoretical spiked concentration. Inter-day precision was evaluated for each QC level on three different days and expressed as %CV. According to the validation guidelines the mean value should be within 15% of the true value, except for the LLOQ where it should be within 20% of the true value.

2.5 Recovery and matrix effect

For recovery, blank urine samples (n=6) were spiked at QC Low and QC High level and were taken through extraction. In parallel, a set of blank matrix samples (n=6) was extracted and spiked after the evaporation step at the QC Low and QC High level. Recovery was expressed as a percentage by comparing the absolute peak areas of the samples spiked before extraction with samples spiked after extraction.

For the IS-corrected matrix effect, a set of blank urine samples (n=6 from three female and three male donors) and a set of samples without matrix (n=6) was taken through extraction. All samples were reconstituted with a solution containing known amounts of the internal standard and analytes at QC Low and QC High levels. Matrix effect was evaluated by comparing peak area ratios in blank matrix samples spiked after extraction with peak area ratios in samples without matrix spiked after extraction.

2.6 Carryover

Carryover was assessed by injecting urine blanks after the highest calibration standard. According to the validation guidelines, carryover should not exceed 20% of the LLOQ.

2.7 Dilution integrity

Highly concentrated samples falling outside the calibration range were diluted. Dilution integrity was assessed by preparing QC dilutions at an appropriate concentration outside the

calibration range and diluting them into the calibration range (n=6). Precision and accuracy were required to be within ± 15% of the target concentration.

2.8 Stability

Long-term stability as well as stability after 6 freeze-thaw (F/T) cycles was assessed at QC Low and QC High levels. Samples were prepared in human urine and were stored for 105 days at -20°C. Freezer and fridge temperatures were monitored and logged daily.

3 Validation results

A method for detection and quantification of mephedrone and five of its Phase I metabolites was validated in human urine.

3.1 Selectivity

No interferences were observed in the extracted blank matrix.

3.2 Linearity

Mean linearity of $r^2 > 0.996$ was achieved for all analytes in all three validation runs.

3.3 LOD and LLOQ

LOD of 0.4 ng/mL (LLOQ of 1.6 ng/mL) for MEPH, HYDROXY, 4-carboxy; 0.15 ng/mL (LLOQ of 0.6 ng/mL) for DHM, NOR; and 0.08 ng/mL (LLOQ of 0.6 ng/mL) for DHNM were achieved in this assay. Table S1 shows calibration parameters for all analytes.

Table S1. LOD, LLOQ, calibration range and calibration parameters for mephedrone and its metabolites in human urine

**Analyte**	**LOD** (ng/mL)	**LLOQ** (ng/mL)	**Range** (ng/mL)	**Intercept ± SD** (n=3)	**Slope ± SD** (n=3)	r^2 **± SD** (n=3)
**MEPH**	0.4	1.6	1.6-50	0.353 ± 0.670	1.97 ± 0.04	0.998 ± 0.001
**DHM**	0.15	0.6	0.6-50	-0.483 ± 0.363	13.8 ± 0.5	1.00 ± 0.00
**NOR**	0.15	0.6	0.6-50	-0.407 ± 0.246	13.0 ± 1.0	0.999 ± 0.001
**HYDROXY**	0.4	1.6	1.6-50	0.068 ± 0.455	4.66 ± 0.47	0.999 ± 0.000
**4-carboxy**	0.4	1.6	1.6-50	-0.844 ± 0.871	4.81 ± 0.57	0.996 ± 0.001
**DHNM**	0.08	0.6	0.6-50	-0.973 ± 0.214	16.2 ± 1.9	0.999 ± 0.000

3.4 Precision and accuracy

Intra-day and inter-day precision and accuracy results, summarised in Table S2, were found to be within the acceptance criteria. The intra-day accuracy was within ± 11.3% of the target concentration while accuracy ranged from 94.7-104% for MEPH, 98.5-107% for DHM, 90.8-102% for NOR, 89.7-104% for HYDROXY, 88.7-103% for 4-carboxy and 94.3-102% for DHNM. The intra-day precision was ≤ 9.39% and ranged from 1.65-5.16% for MEPH, 1.44-6.61% for DHM, 0.978-4.18% for NOR, 1.89-5.23% for HYDROXY, 4.15-9.39% for 4-carboxy and 0.956-6.21% for DHNM. Inter-day precision and accuracy results were acceptable over the validated range with %CV ≤ 7.63% and accuracy within ± 7.50% of the target concentration.

*Table S2. Precision and accuracy at QC Low, QC Med and QC High for all analytes in human urine; * average value of 18 measurements over 3 days*

**Analyte**	**True value** (ng/mL)	**Mean** (ng/mL), **%CV, % accuracy**			
		**Day 1 n=6**	**Day 2 n=6**	**Day 3 n=6**	**Inter-day n=18 ***
**MEPH**	2	1.89<br>3.95%<br>94.7%	2.02<br>4.76%<br>101%	2.01<br>4.50%<br>100%	1.97<br>5.14%<br>98.7%
	10	9.83<br>3.34%<br>98.3%	9.95<br>5.16%<br>99.5%	10.4<br>1.65%<br>104%	10.0<br>4.11%<br>100%

	40	38.0<br>3.60%<br>94.9%	38.2<br>2.22%<br>95.4%	39.5<br>1.91%<br>98.8%	38.6<br>3.12%<br>96.4%
**DHM**	1	1.03<br>5.15%<br>103%	1.07<br>1.44%<br>107%	1.02<br>3.62%<br>102%	1.04<br>4.02%<br>104%
	10	10.3<br>4.35%<br>103%	10.4<br>3.79%<br>104%	10.6<br>6.61%<br>106%	10.4<br>4.89%<br>104%
	40	39.5<br>6.27%<br>98.8%	41.2<br>3.05%<br>103%	39.4<br>2.13%<br>98.5%	40.0<br>4.48%<br>100%
**NOR**	1	0.908<br>2.81%<br>90.8%	0.968<br>2.16%<br>96.8%	0.974<br>3.00%<br>97.4%	0.950<br>4.09%<br>95.0%
	10	10.1<br>4.18%<br>101%	10.2<br>1.65%<br>102%	9.70<br>0.978%<br>97.0%	9.99<br>3.26%<br>99.9%
	40	38.6<br>3.79%<br>96.5%	40.0<br>1.66%<br>100%	40.1<br>2.38%<br>100%	1.23<br>3.12%<br>99.0%
**HYDROXY**	2	2.01<br>4.13%<br>101%	1.91<br>2.98%<br>95.4%	1.92<br>2.04%<br>95.9%	1.95<br>3.87%<br>97.3%
	10	10.1<br>3.31%<br>101%	10.4<br>3.50%<br>104%	9.62<br>2.68%<br>96.2%	10.1<br>4.51%<br>101%
	40	38.7<br>5.23%<br>96.7%	41.0<br>1.89%<br>102%	35.9<br>2.82%<br>89.7%	38.5<br>6.47%<br>96.3%
**4-carboxy**	2	2.02<br>8.71%<br>101%	1.88<br>4.59%<br>94.2%	2.06<br>5.59%<br>103%	1.99<br>7.32%<br>99.4%
	10	9.39<br>4.46%<br>93.9%	9.98<br>9.39%<br>99.8%	9.67<br>4.15%<br>96.7%	9.68<br>6.69%<br>96.8%
	40	36.5<br>6.76%<br>91.1%	39.0<br>6.14%<br>97.5%	35.5<br>7.61%<br>88.7%	37.0<br>7.63%<br>92.5%
**DHNM**	1	0.943<br>5.48%<br>94.3%	0.985<br>2.61%<br>98.5%	0.963<br>3.11%<br>96.3%	0.964<br>0.920%<br>96.4%
	10	10.0<br>4.29%<br>100%	10.2<br>5.31%<br>102%	9.62<br>2.68%<br>96.2%	10.1<br>0.461%<br>101%
	40	39.5<br>6.21%	406<br>1.16%	38.6<br>0.956%	39.6<br>1.52%

	98.7%	101%	96.5%	98.8%

3.5 Recovery and matrix effect

As shown in Table S3, recovery was found to be greater than 74.7 ± 3.8% for all analytes, except 4-CARBOXY for which recovery was 35.8 ± 7.2% at QC Low and 34.3 ± 6.3% at QC High. Reasons for such low recovery were explained in our recent publication [1]. Even though it is recommended for recovery to be greater than 50% [2], desired sensitivity as well as acceptable precision and accuracy were achieved for 4-CARBOXY (Table S2).

IS-corrected matrix effect values were within ± 20.4% at both QC levels, except for HYDROXY which was suppressed by 34.4 ± 7.4% at QC Low and by 32.6 ± 4.6% at QC High (Table S3). This may be due to the lack of matching deuterated IS which is currently not commercially available. However, assay precision and accuracy for HYDROXY at QC Low and QC High were within the acceptable limits (Table S2).

Table S3. Analyte recovery and matrix effect at QC Low and QC High in human urine

Analyte	Recovery (%CV), n=6		Matrix Effect (%CV), n=6	
	QC Low	QC High	QC Low	QC High
**MEPH**	91.7% (10.4%)	82.6% (5.6%)	98.3% (5.0%)	95.5% (0.8%)
**DHM**	79.5% (6.0%)	82.4% (5.3%)	101% (1.0%)	98.6% (0.4%)
**NOR**	74.7% (3.8%)	79.0% (5.4%)	93.2% (1.2%)	90.9% (0.5%)
**HYDROXY**	84.3% (6.1%)	87.2% (6.0%)	65.6% (7.4%)	67.4% (4.6%)
**4-carboxy**	35.8% (7.2%)	34.3% (6.3%)	80.3% (6.9%)	79.6% (5.0%)
**DHNM**	90.3% (5.4%)	97.7% (5.5%)	91.4% (3.0%)	88.6% (1.1%)

3.6 Carryover

Carryover was not observed.

3.7 Dilution integrity

All analytes showed good precision and accuracy following 1 in 100 and 1 in 1000 dilutions in human urine. For 1 in 100 dilution, %CV ranged from 4.4-11.0% and accuracy was within ± 6.0% of the target concentration (Table S4). For 1 in 1000 dilution, %CV ranged from 2.4-6.6% and accuracy was within ± 7.0% of the target concentration (Table S5).

Table S4. Dilution integrity (1 in 100) for mephedrone and its metabolites in human urine

Analyte	**1 in 100 Dilution** (n=6)			
	**True value** (ng/mL)	**Calculated mean** (ng/mL)	**%CV**	**% accuracy**
**MEPH**	10	9.98	4.4%	99.8%
**DHM**		10.0	6.3%	100%
**NOR**		9.75	6.4%	97.5%
**HYDROXY**		10.2	11.0%	102%
**4-carboxy**		10.6	10.7%	106%
**DHNM**		9.82	8.6%	98.2%

Table S5. Dilution integrity (1 in 1000) for mephedrone and its metabolites in human urine

Analyte	**1 in 1000 Dilution** (n=6)			
	**True value** (ng/mL)	**Calculated mean** (ng/mL)	**%CV**	**% accuracy**
**MEPH**	10	9.90	2.4%	99.0%
**DHM**		10.3	3.8%	103%
**NOR**		9.30	5.2%	93.0%
**HYDROXY**		9.44	3.5%	94.4%
**4-carboxy**		9.73	6.6%	97.3%
**DHNM**		9.57	3.9%	95.7%

3.8 Stability

Long term stability of mephedrone and its metabolites in human urine was assessed following storage for 105 days at -20°C at QC Low and QC High. As shown in Table S6, all analytes were

within ± 15% of their initial concentration under the investigated conditions, except for DHNM at QC Low as well as HYDROXY and 4-carboxy at QC High which lost 61.2 ± 2.9%, 33.4 ± 4.1% and 43.2 ± 8.9%, respectively.

In a separate experiment, stability samples were taken through 6 freeze-thaw (F/T) cycles from -20°C to room temperature to assess stability following sample thawing which may be required for sample re-analysis due for example to a failed batch or the need for sample dilution. All analytes were stable following 6 F/T cycles, except 4-carboxy at QC High and DHNM at QC Low, which lost 17.2 ± 5.5% and 18.8 ± 5.3%, respectively.

Table S6. Long term and freeze-thaw stability of mephedrone and its metabolites in human urine following storage at -20°C

**Analyte**	**Long term stability (%CV)**, n=6		**F/T stability (%CV)**, n=6	
	**QC Low**	**QC High**	**QC Low**	**QC High**
**MEPH**	101% (2.5%)	99.3% (1.3%)	102% (2.6%)	93.8% (0.8%)
**DHM**	92.0% (1.7%)	102% (0.7%)	100% (1.8%)	98.5% (1.4%)
**NOR**	102% (2.4%)	90.9% (1.2%)	97.5% (2.0%)	86.4% (1.9%)
**HYDROXY**	89.3% (2.7%)	66.6% (4.1%)	102% (3.2%)	90.2% (3.4%)
**4-carboxy**	94.6% (1.5%)	56.8% (8.9%)	103% (6.9%)	82.8% (5.5%)
**DHNM**	38.8% (2.9%)	90.3% (1.1%)	81.2% (5.3%)	97.9% (1.1%)

4 DHNM synthesis

DHNM was successfully synthesized (yield: 51%). Formula $C_{10}H_{16}NO^+$; HRMS [M + H^+] calculated m/z 166.1226, observed 166.1227 (+0.001 ppm); observed MS/MS fragments with collision energy 20 eV were consistent with those reported in the literature [3]. ^1H NMR ($CDCl_3$): δ 7.22 (d, J = 8.0 Hz, 2H, Ar-H), 7.16 (d, J = 8.0 Hz, 2H, Ar-H), 4.50 (d, J = 4.0 Hz, 1H, CH (OH)), 3.19 (br, 1H, CH (CH_3)), 2.35 (s, 3H, Ar-CH_3) and 0.98 (d, J = 8.0 Hz, 3H, CH (CH_3)). ^1H NMR data (see Figure S1) is consistent with the literature except for the signal at 3.19 ppm being previously reported as a multiplet [4].

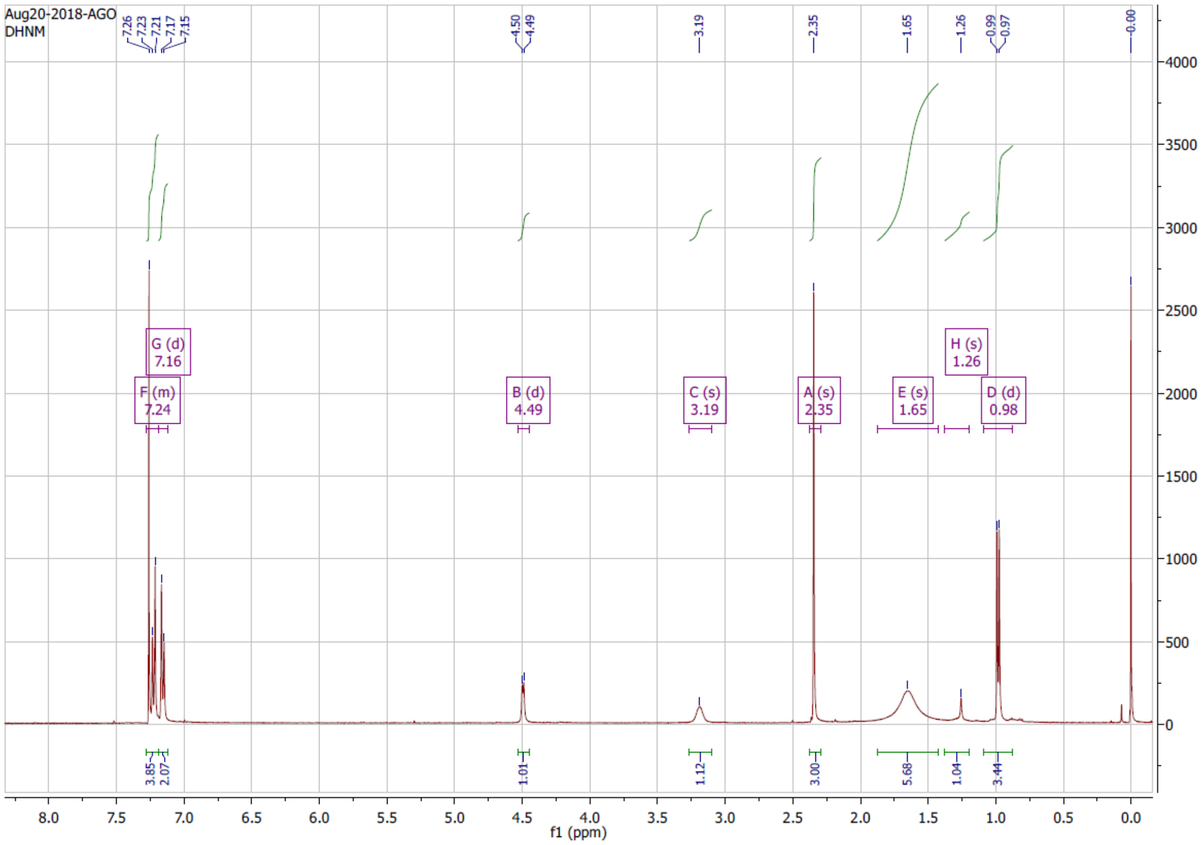

Figure S1. ^1H NMR spectrum of DHNM

5 References

1. Czerwinska J, Parkin MC, Dargan PI, George C, Kicman AT, Abbate V. Stability of mephedrone and five of its phase I metabolites in human whole blood. *Drug Test. Anal.* **2018**;*11*:586–594.
2. Peters FT, Drummer OH, Musshoff F. Validation of new methods. *Forensic Sci. Int.* **2007**;*165*:216–224.
3. Pozo OJ, Ibanez M, Sancho JV, et al. Mass spectrometric evaluation of mephedrone in vivo human metabolism: identification of phase I and phase II metabolites, including a novel succinyl conjugate. *Drug Metab. Dispos.* **2014**;*43*:248–257.
4. Brandt SD, Baumann MH, Partilla JS, et al. Characterization of a novel and potentially lethal designer drug (±)-cis-para-methyl-4-methylaminorex (4,4'-DMAR, or 'Serotoni'). *Drug Test. Anal.* **2014**;6:684–95.
